# Right Ventricular Phenotyping Can Lead to Pulmonary Vascular Therapy Response in Those with Pulmonary Hypertension with COPD: A Single-Center Cohort Study

**DOI:** 10.3390/jcdd12090366

**Published:** 2025-09-18

**Authors:** Oluwafeyijimi Salako, Abhishek Singh

**Affiliations:** 1 Department of Cardiology, Gagnon Cardiovascular Institute, Morristown Medical Center, Morristown, NJ 07960, USA; 2 Heart Success Program (Advanced Heart Failure Program), Department of Cardiology, Gagnon Cardiovascular Institute, Morristown Medical Center, Morristown, NJ 07960, USA

**Keywords:** sildenafil, inhaled treprostinil, pulmonary vascular disease

## Abstract

Pulmonary hypertension (PH) with chronic obstructive pulmonary disease (COPD) is associated with poor survival with no approved therapies. We report on the response to inhaled treprostinil (iTRE) of a small retrospective cohort of PH-COPD patients with a baseline “PH-right ventricular (RV) phenotype”, defined by a RV-dependent circulatory limitation derived from a combination of echocardiographic and hemodynamic criteria. Patients were started on inhaled treprostinil with significant improvement in six-minute walk distance, NT-proBNP, and improved RV metrics by echocardiography. The preliminary findings of this cohort provide evidence for the importance of precision phenotyping of PH-COPD.

## 1. Introduction

Pulmonary hypertension (PH) associated with chronic obstructive pulmonary disease (COPD) is associated with decreased quality of life, functional status, and survival [1,2,3,4,5,6]. Approximately 1–5% of COPD patients develop severe pulmonary hypertension (PH), defined as a mean pulmonary artery pressure (mPA) of ≥35 mmHg, a mPA of ≥25 with a cardiac index (CI) of <2 L/min/m^2^, or a pulmonary vascular resistance (PVR) of >5 WU [7,8].

There have been numerous studies evaluating various pulmonary arterial hypertension (PAH) therapies in PH-COPD patients, with registry data and two meta-analyses of these trials suggesting a treatment benefit, specifically with sildenafil at no significant expense to gas exchange [6,9,10,11,12,13]. Inhaled treprostinil (iTRE) is an attractive agent in PH-lung disease patients due to the decreased theoretical risk of V/Q mismatch. While efficacious in PH–interstitial lung disease (PH-ILD), the PERFECT study was terminated early by the data and safety monitoring committee based on the totality of evidence that iTRE exposure increased the risk of serious adverse events in a PH-COPD population [14,15]. However, in subgroup analysis, 7/66 patients with a baseline mPA pressure of ≥40 mmHg, FEV1 of ≥40%, and diffusion capacity of the lung for carbon monoxide (D_LCO_) of >25% showed evidence of a potential response [16]. We report here on a cohort of PH-COPD patients with significant improvement in their six-minute walk distance (6MWD) and N-terminal pro-B-type natriuretic peptide (NT-proBNP) with concordant RV morphometric improvements by echocardiography (TTE) on iTRE therapy phenotyped for a baseline right ventricular (RV)-dependent circulatory limitation by TTE and right heart catheterization (RHC).

## 2. Materials and Methods

**Figure 1:**
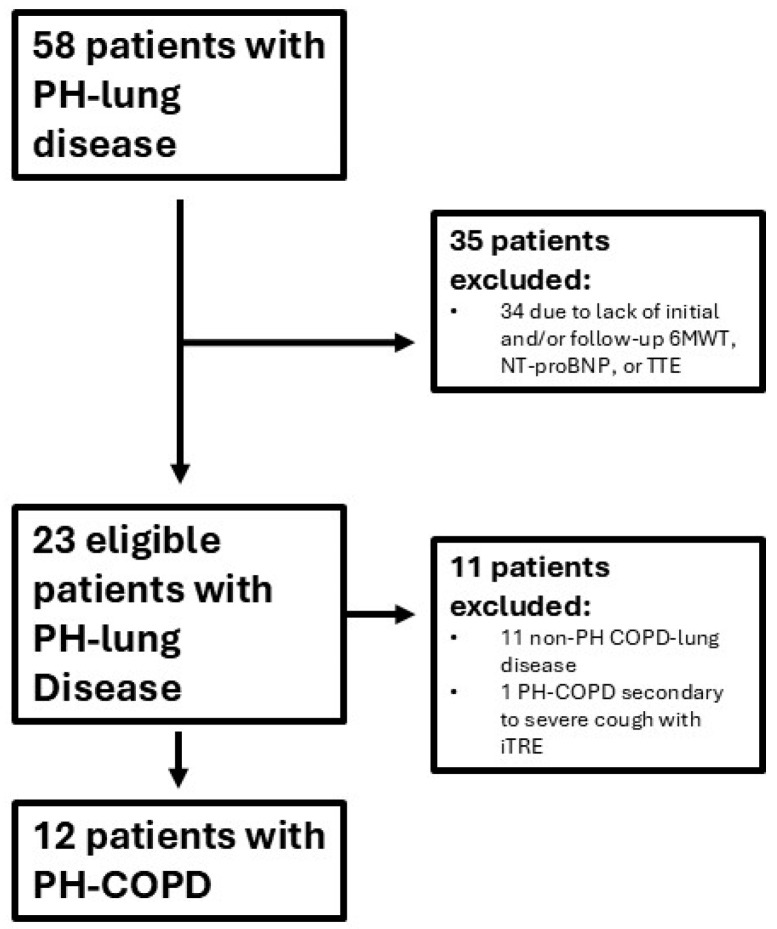
Enrollment criteria. Fifty-eight subjects with pulmonary hypertension with lung disease underwent right heart catheterization between October 2020 and December 2024. Twenty-three subjects were eligible for study inclusion. Of these, twelve subjects were included in the PH-COPD cohort.

We retrospectively analyzed and included 12 PH-COPD patients referred to and treated at a large pulmonary hypertension referral center on iTRE therapy that met the following five criteria from November 2020 to October 2024 (Figure 1): (1) FEV_1_/FVC < 70 and FEV_1_ < 80 (Criteria A) or FEV_1_ < 80% with emphysema by CT based on radiologist and referring pulmonologist reads (Criteria B) [17,18,19]; (2) mPA ≥ 25 and PVR > 4; (3) baseline TTE consistent with a “PH-RV phenotype” defined by a combination of systolic septal flattening and/or right ventricular outflow track (RVOT) pulse wave Doppler notching with any of the following: hypertrophy, dilation, and/or reduced function [20]; (4) a complete pre-treatment data set including spirometry with D_LCO_, RHC, CT scan, TTE, six-minute walk test, and NT-proBNP; and (5) included patients had repeat TTE, NT-proBNP, and six-minute walk test completed within six months of maximum tolerated pulmonary arterial hypertension (PAH) therapy. All TTE images were independently reviewed in a blinded fashion by the senior author. Patients were excluded if they had Group 1, 2, 4, or 5 pulmonary hypertension or evidence of ILD or combined pulmonary fibrosis and emphysema (CPFE). Six of the twelve patients, subgroup A, met criteria A, and the other six, subgroup B, met criteria B for inclusion. PAH therapy was initiated within one month of hemodynamic assessment. Baseline characteristics are presented as mean +/− SD for continuous variables with ranges as appropriate and as frequencies and proportions (%) for categorical variables. Comparisons among baselines and among treatments were conducted using paired and unpaired *t*-test for continuous variables. Chi-square or Fischer’s test was used for categorical variables where appropriate. All patients gave written informed consent for all procedures. The study protocol was reviewed by the Atlantic Health System institutional review board and found to be exempt from the regulations that govern human subject research (IRB submission # 2249315-1).

Generative artificial intelligence (GenAI) has not been used in this paper.

## 3. Results

Baseline clinical features of our cohort were as follows (Table 1): age (years), 73.8 +/− 7.9 (range 57–85); female, 58%; and BMI (kg/m^2^), 24 +/− 5.4 (range 16.5–33.7). Comorbidity burden was as follows: coronary artery disease, 41.7%; hypertension, 66.7%; hyperlipidemia, 66.7%; diabetes, 25%; atrial fibrillation, 25%; and obstructive sleep apnea, 50%. All patients were on iTRE (mcg) on a mean dose of 75.2 +/− 27.6 (min. 36, max. 126 per dose; 12 of 12 patients). Those on sildenafil were on a mean dose (mg) of 44 +/− 22.7 q8hrs (min. 20, max. 80 per dose; 10 of 12 patients).

All included patients carried a diagnosis of COPD on supplemental O_2_ at a flow rate (L/min) of 3.4 +/− 1.4 (2–5 L/min). All patients were on stable optimized background inhaler therapy, including LABA (1/12), LABA/LAMA (2/12), LABA/ICS (5/12), and LABA/LAMA/ICS (4/12). Within subgroup A, four patients were on LABA/LAMA/ICS therapy, and one each on LABA/ICS and LABA inhaler therapy. The combined FEV_1_ (%) was FEV_1_ 65 +/− 12.8 (39–80), FVC (%) 83.8 +/− 19.9 (57–127), FEV_1_/FVC (%) 70.5 +/− 17.8 (48–95), and D_LCO_ (%) 30.8 +/− 9.5 (17–46). All twelve patients had emphysema by imaging, with eight of twelve having evidence of moderate or severe emphysema. Four of six, in subgroups A and B each, had moderate or severe parenchymal lung disease. The PFTs for subgroup A were FEV_1_ (%) 59.2 +/− 14.5, FVC (%) 85 +/− 17.3, FEV_1_/FVC (%) 58.2 +/− 7.1, and D_LCO_ (%) 28 +/− 7.2 (range 18–46). The PFTs for subgroup B were FEV_1_ (%) 70.8 +/− 8.3, FEV (%) 82 +/− 23.8, FEV_1_/FVC (%) 82.8 +/− 16.8, and D_LCO_ (%) 33.7 +/− 11.7.

Rest hemodynamics revealed the following: RAP (mmHg) 8.5 +/− 6.8, mPA (mmHg) 42.6 +/− 10, end-expiratory PCWP (mmHg) 11.2 +/− 3.5, cardiac output (CO, L/min) 3.4 +/− 1.1, cardiac index (L/min/m^2^) 1.9 +/− 0.6, PVR (WU) 10.2 +/− 4.4, and pulmonary artery compliance (mL/mmHg) 1.2 +/− 0.6. Eleven of the twelve patients had a mPA pressure of >35 mmHg or mPA of >25 mmHg with a CI of <2.2.

**Figure 2:**
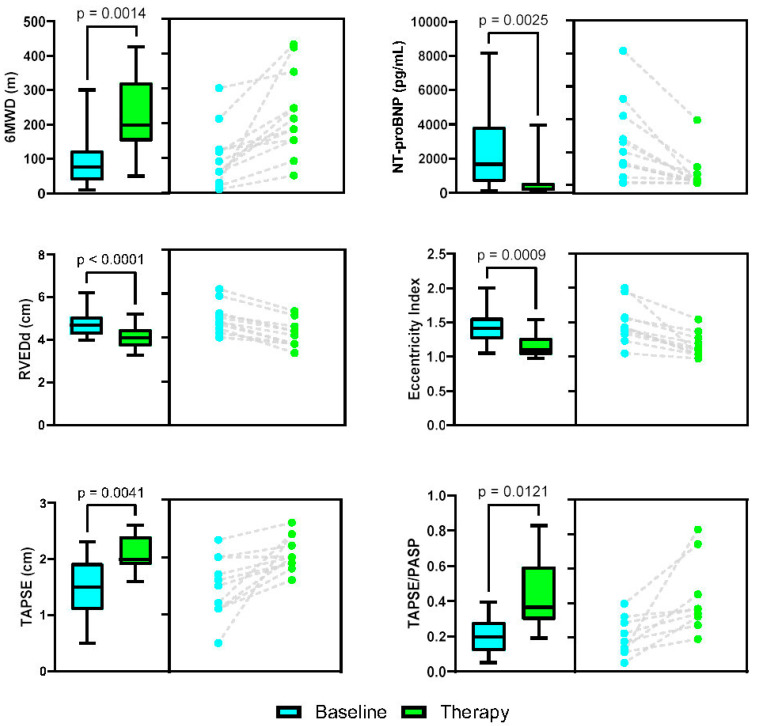
PH-COPD pre-treatment (blue) and on max. tolerated PAH therapy (green). Each pair of graphs is of the same data set with the Piston plot on left (data graphically depicted as mean +/− SD) with the combined cohort and before and after plot on right of each individual patient data point change. 6MWD, six-minute walk distance; NT-proBNP, N-terminal pro-brain natriuretic peptide; RVEDd, basal right ventricular end-diastolic dimension; TAPSE, tricuspid annular plane systolic excursion; TAPSE/PASP; tricuspid annular plane systolic excursion/pulmonary artery systolic pressure.

Key metrics are shown in Figure 2. All patients had a LVEF of >55%. TTE features comparing pre-treatment to maximum tolerated therapy included metrics of RV reverse remodeling and improved ventricular interdependence based on the decrease in basal RV end-diastolic dimension (cm) from 4.8 +/− 0.67 to 4.1 +/− 0.59 cm (*p* < 0.0001) and decrease in LV systolic eccentricity index from 1.46 +/− 0.29 to 1.15 +/− 0.17 (*p* < 0.0009), respectively. RV functional metrics improved with increased TAPSE (cm) from 1.47 +/− 0.50 to 2.1 +/− 0.30 (*p* < 0.0041) and improved RV-PA coupling with increased TAPSE/PASP ratio 0.20 +/− 0.10 to 0.43 +/− 0.21 (*p* < 0.0121). There was increased biventricular stroke volume based on the increase in RVOT velocity time integral (VTI, mm) from 11.2 +/− 4.5 to 15.6 +/− 2.5 (*p* < 0.02) and left ventricular outflow tract (LVOT) VTI (mm) from 17.3 +/− 4.9 to 22.2 +/− 2.8 (*p* < 0.0093), suggestive of improved global circulatory function. This was accompanied by the transition from a mid-systolic RVOT pulse wave Doppler notch in 92% of patients to late-systolic notch in 67% of patients (*p* < 0.036), a noninvasive surrogate for decreased PVR and improved pulmonary vascular compliance based on the decrease in the velocity of pulmonary vascular wave reflection [21,22].

All patients reported subjective mild improvements in shortness of breath on sildenafil therapy, but a dose-dependent improvement in symptoms and walk distance with an up-titration of iTRE. Diuretic therapy was decreased in 5 of 12 patients and remained the same in the other 7. One patient who did not tolerate iTRE therapy due to adverse side effects at the lowest dose was not included in the study (Figure 1).Three of the twelve patients required down-titration of sildenafil and iTRE due to worsening oxygenation. No arterial blood gas (ABG) data was available to assess for V/Q mismatch. Despite this, all patients showed improvements in their 6MWD (m) on a maximum tolerated stable dose of iTRE from 101.1 +/− 84.6 m to 235.8 +/− 121.8 (*p* < 0.0011) and NT-proBNP from 2471.8 +/− 2381.2 to 620.1 +/− 1087.7 (*p* < 0.0014).

## 4. Discussion

To our knowledge, this is the first report of a significant functional improvement in a PH-COPD cohort on iTRE and sildenafil therapy with improved 6MWD and decreased NT-proBNP supported by improved RV metrics on serial TTE monitoring. Despite the small sample size, each patient showed improvements in the reported parameters from baseline to maximum tolerated pulmonary vascular therapy. The included patients were enriched for a COPD pulmonary vascular phenotype with a mPA pressure elevation of ≥30 in 11 of 12 patients and a PVR of >5 WU in all patients with a “PH-RV phenotype” by TTE and reduced CO/CI by RHC.

While it is possible that the included patients had pulmonary vascular disease with concurrent COPD, eight of twelve patients had moderate to severe parenchymal lung disease by lung imaging, and subgroup A was comparable to the cohort in the “responder” group of the PERFECT trial with moderate to severe COPD by GOLD criteria with similar hemodynamics [16]. In comparison with the “responder” group that showed a ≥15% increase in 6MWD and ≥20% reduction in NT-proBNP in the PERFECT study, the response to iTRE was significantly more pronounced in our cohort. A comparison of potential iTRE “responders” to adverse responders in the PERFECT study noted findings suggestive of greater circulatory limitation with a higher mPA, higher PVR, and ~5x higher NT-proBNP, which is in line with our findings [16].

The primary reason for a positive response to therapy in our cohort may be attributable to the use of TTE to screen for concurrent HFpEF and to identify a “PH-RV phenotype” with resultant RV-dependent circulatory limitation. During RHC, the use of mean end-expiratory PCWP through multiple cardiac cycles decreased the likelihood of the underestimation of PCWP, making HFpEF less likely to be present in our cohort. Despite the presence of HFpEF risk factors, the above methodology led to a stable to decreased diuretic requirement, suggestive of improved circulatory function. Coupling TTE-based morphometric assessment and invasive hemodynamics may allow for an improved phenotyping of these patients. As noted by the investigators in the PERFECT study, TTE data was not collected, and significant variability in the method of PCWP measurement may have resulted in the enrollment of patients with concurrent HFpEF. Additional reasons for a positive response may include an additive effect of PDE5i and iTRE therapy based on acute hemodynamic improvements seen with sequential single dose of sildenafil followed by iTRE [23]. Finally, the average iTRE dosing was higher in our cohort compared with the PERFECT study, ranging from 36 mcg to 126 mcg, with improved 6MWD in the entire dosing range making this less likely to be the main differentiator.

Despite the significant benefit, three patients required down-titration of pulmonary vascular therapy due to increased shortness of breath raising potential concerns with the up-titration of therapy. The use of higher average doses of sildenafil and iTRE in our study compared with prior studies may have increased this risk as sildenafil at lower doses has been shown to have minimal negative gas exchange effects [6]. The lack of ABG data precludes the ability to comment on V/Q mismatch as the etiology. Alternatively, left heart limiting physiology, either via left heart non-compliance or “over-treatment” of the pulmonary vasculature, may result in increased shortness of breath. Right heart catheterization was not performed with an intra-procedure administration of therapy to exclude this possibility.

The limitations of our study include its retrospective single-center nature, small sample size, and referral bias, as well as not having a control comparator group. The study is limited by lack of follow up PFTs on iTRE therapy and ABG analysis to assess their effect on lung function pre- and post-therapy and V/Q matching, respectively. While a larger study is needed, our results suggest that coupling TTE-based morphometric assessment and invasive hemodynamics in PH-COPD patients allows for selection of those with an RV-dependent circulatory limitation that may derive benefit from PAH therapy when the cardiac limitation is greater than the pulmonary limitation.

## Figures and Tables

**Table 1:** Baseline patient demographic data and functional parameters.

Characteristics				
Total number of patients	COPD (*n* = 12)			
Female	7 (58.0%)			
Age (Years)	73.8 (57–85)			
**Comorbidities ^a^**				
BMI (kg/m^2^)	24 (16.5–33.7)			
CAD	5 (41.7%)			
HTN	8 (66.7%)			
DM II	3 (25.0%)			
Atrial fibrillation/Flutter	3 (25.0%)			
OSA	6 (50.0%)			
**Pulmonary Data**				
	Combined	Subgroup A	Subgroup B	***p* value**
	*n* = 12	*n* = 6	*n* = 6	
FEV_1_ (%)	65 +/− 12.8 (39–80)	59.2 +/− 14.5	70.8 +/− 8.3	0.1175
FVC (%)	83.8 +/− 19.9 (57–127)	85 +/− 17.3	82 +/− 23.8	
FEV_1_/FVC (%)	70.5 +/− 17.8 (48–95)	58.2 +/− 7.1	82.8 +/− 16.8	0.0079
D_LCO_ (%)	30.8 +/− 9.5 (17–46)	28 +/− 7.2	33.7 +/− 11.7	0.3251
Supplemental O_2_ (L/min)	12 (100%) 3.4 +/− 1.4 (2–5 L/min)			
Emphysema on CT Chest	12 (100%)	6 (50%)	6 (50%)	
Mild	4 (33.3%)	2 (33%)	2 (33%)	
Moderate to severe	8 (66.7%)	4 (67%)	4 (67%)	
**Echocardiographic Parameters**				
	**Pre—Treatment** **Mean ± STDev**	**On—Treatment** **Mean ± STDev**		***p* value**
RVEDd (cm)—basal width	4.8 +/− 0.67	4.1 +/− 0.59		<0.0001
LV systolic EI index	1.46 +/− 0.29	1.15 +/− 0.17		<0.0009
TAPSE (cm)	1.47 +/− 0.50	2.1 +/− 0.30		<0.0041
TAPSE/PASP ratio	0.20 +/− 0.10	0.43 +/− 0.21		<0.0121
RVOT VTI (cm)	11.2 +/− 4.5	15.6 +/− 2.5		<0.02
LVOT VTI (cm)	17.3 +/− 4.9	22.2 +/− 2.8		<0.0093
RVOT PW Doppler notching				
Mid-systolic notch	11 (92%)	4 (33%)		<0.036
Late-systolic notch	1 (8%)	8 (67%)		

Notes: For nominal variables *n* (%) and for continuous variables’ mean (range) standard deviation as applicable. Abbreviations: BMI, body mass index; CAD, coronary artery disease; HTN, hypertension; DM II, diabetes mellitus type 2; OSA, obstructive sleep apnea; RVEDd, right ventricular end-diastolic diameter; LV systolic EI, left ventricular systolic eccentricity index; RVOT, right ventricular outflow tract; LVOT, left ventricular outflow tract; VTI, velocity time integral; PW Doppler, pulse wave Doppler; TAPSE, tricuspid annular plane systolic excursion; PASP, pulmonary artery systolic pressure. ^a^ The co-morbidities are based on chart review.

## Data Availability

Data supporting this study are available upon reasonable request. Please contact the corresponding author.
